# Decoding identity from motion: how motor similarities colour our perception of self and others

**DOI:** 10.1007/s00426-020-01290-8

**Published:** 2020-02-06

**Authors:** Alexandre Coste, Benoît G. Bardy, Stefan Janaqi, Piotr Słowiński, Krasimira Tsaneva-Atanasova, Juliette Lozano Goupil, Ludovic Marin

**Affiliations:** 1 EuroMov Digital Health in Motion, Univ. Montpellier, IMT Mines Alès, Montpellier, France; 2 Department of Mathematics and Living Systems Institute, Translational Research Exchange @ Exeter, College of Engineering, Mathematics and Physical Sciences, University of Exeter, Exeter, EX4 4QF UK; 3 Department of Mathematics and Living Systems Institute, College of Engineering, Mathematics and Physical Sciences, University of Exeter, Exeter, EX4 4QF UK; 4 EPSRC Centre for Predictive Modelling in Healthcare, University of Exeter, Exeter, EX4 4QJ UK

## Abstract

For more than 4 decades, it has been shown that humans are particularly sensitive to biological motion and extract socially relevant information from it such as gender, intentions, emotions or a person’s identity. A growing number of findings, however, indicate that identity perception is not always highly accurate, especially due to large inter-individual differences and a fuzzy self-recognition advantage compared to the recognition of others. Here, we investigated the self-other identification performance and sought to relate this performance to the metric properties of perceptual/physical representations of individual motor signatures. We show that identity perception ability varies substantially across individuals and is associated to the perceptual/physical motor similarities between self and other stimuli. Specifically, we found that the perceptual representations of postural signatures are veridical in the sense that closely reflects the physical postural trajectories and those similarities between people’ actions elicit numerous misattributions. While, on average, people can well recognize their self-generated actions, they more frequently attribute to themselves the actions of those acting in a similar way. These findings are consistent with the common coding theory and support that perception and action are tightly linked and may modulate each other by virtue of similarity.

**Electronic supplementary material:**

The online version of this article (10.1007/s00426-020-01290-8) contains supplementary material, which is available to authorized users.

## Introduction

One of the more stunning examples of the human visual system ability is to ‘decode’ the identity of individuals directly through their motion even from impoverished point-light animations (Beardsworth & Buckner, 1981; Cutting & Kozlowski, 1977; Loula, Prasad, Harber & Shiffrar, 2005; Yovel & O’Toole, 2016). Such an ability requires fine perceptual information reflecting the subtle nuances in the way people move as well as a certain robustness against intra-individual variations such as viewpoint changes, inter-trials variability or affect (e.g., Jokisch, Daum & Troje, 2006; Pilz & Thornton, 2017; Prasad & Shiffrar, 2009). While it has long been shown the importance of familiarity through repeated exposure to cope with the real-world within-person variability (Beardsworth & Buckner, 1981; Cutting & Kozlowski, 1977; Hohmann, Troje, Olmos & Munzert, 2011; Loula et al., 2005; Repp & Knoblich, 2004), it was only recently that the existence of a person-specific motion signature—a sort of kinematic fingerprint—has been empirically demonstrated (e.g., Hart, Noy, Feniger-Schaal, Mayo & Alon, 2014; Słowiński et al., 2016). In theory, the individual motor signature is supposed to be largely stable across time (invariance) and different from those of others (distinctiveness). In fact, dynamic laws of motion shared by all human beings strongly constrain body motion and lead inevitably to low inter-individual variations. Thus, behavioural traits including gait and gestures are occasionally qualified as soft biometrics due to their lack of distinctiveness and low reliability compared to physiological-based measurements such as fingerprints or DNA matching (Dantcheva, Velardo, D’angelo & Dugelay, 2011; Steel, Ellem & Baxter, 2015). They remain, however, commonly used alongside other cues (e.g., faces) and have the advantage of being accessible from a distance and do not involve the subject cooperation (Rice, Phillips, Natu, An & O’Toole, 2013). Moreover, recognition accuracy can be enhanced using less constrained actions such as dancing or boxing to promote the uniqueness of person’s motion signature (Loula et al., 2005). Some movement styles may also facilitate the identification of individuals, whereas others may make it more difficult (Koul, Cavallo, Ansuini & Becchio, 2016). For instance, skilled performers such as musicians, dancers or athletes often have more individualistic movement styles providing rich kinematic patterns available for recognition (Calvo-Merino, Glaser, Grèzes, Passingham & Haggard, 2005; Repp & Knoblich, 2004). Similarly, it has been shown that distinctiveness or caricature may enhance identification (e.g., Lee, Byatt & Rhodes, 2000). In the same way as distinctive faces are easier to recognize than more typical faces (e.g., Light, Kayra-Stuart & Hollander, 1979), the manipulation of kinematic pattern by exaggerating temporal (Hill & Pollick, 2000) or spatial properties (Pollick, Fidopiastis & Braden, 2001) of a biological movement can enhance identity recognition performance. Otherwise, identity recognition from point-light displays is not always highly accurate and the conditions leading to successful recognition remain largely unclear. Recognition rates for self-recognition vary between 40% and 100% depending on the study (Beardsworth & Buckner, 1981; Cutting & Kozlowski, 1977; Wolff, 1931). Large inter-individual differences are also generally reported and are not fully explained. For instance, Beardsworth and Buckner (1981) reported differences in the participants’ ability to recognize themselves and others and that some subjects were easier to recognize than others. One currently unexplored explanation path is linked to motion similarity. Similarity effects on perceptual recognition tasks are indeed predicted by common coding theory of perception and action and the related psychological studies of pattern and form recognition. According to common coding theory (Prinz, 1997; Hommel, Müsseler, Aschersleben & Prinz, 2001), perception and action are tightly linked and may modulate each other by virtue of similarity. Perceiving or performing an action results in the activation of the same representations (i.e., common codes) and the degree of activation is assumed to vary depending on whether the observed actions are in a similar way as one would perform them oneself, or in a different way. Since each individual can best reproduce its own actions, the perceived similarity as well as the activation of action representation are assumed to be maximal and might explain a greater performance for self-recognition despite the fact that people are not used to observing themself from an external point of view (Beardsworth & Buckner, 1981; Cook, Johnston & Heyes, 2012; Jokish, Daum & Troje, 2006; Knoblich & Flach, 2001; Loula et al., 2005; Prasad & Shiffrar, 2009). Yet, it raises the problem of action attribution in particular if a person moves the same way as ours. One would, therefore, expect that an increase in overlap between self-generated actions and other-generated actions should cause attribution errors. The related studies of pattern and form recognition often assume that the greater the similarity between a pair of stimuli, the more likely one will be confused with the other one (e.g., Luce, 1963; Tversky & Gati, 1982; Ashby & Perrin, 1988). Since the perception of moving shapes is derived from the perception of static forms (Cutting & Kozlowski, 1977), one would expect the same effect. Finally, the similarity issue between individuals is also problematic from an algorithmic point of view. Biometric systems compute the similarity between the input biometric signature (i.e., features set extracted from data) and the signatures previously stored in the database (Jain, Ross & Nandakumar, 2011). If the similarity measure exceeds a threshold, then a “match” or an “accept” is declared, if not, then a “non-match” or a “reject” occurs. However, when the biometric signatures of two individuals are very similar, errors occur (false accept or false reject).

In the present study, we examined the extent to which motor similarities mediate identity perception. To this end, we first recorded the idiosyncratic motion variability of individuals (i.e., individual motor signatures) in a postural improvisation task. Next, using point-light depictions of themselves and fourteen other people (unknown to the participants) with a wide range of signatures (similar/different), we probed the self/other discrimination performance.

## Methods

### Participants

Twenty healthy volunteers (12 males, 8 females; mean age = 22.6 years, SD = 1.7; mean height = 173 cm, SD = 7.5; mean weight = 69.4 kg, SD = 11.9) participated in the study. Five of them were excluded from the analysis, since they did not complete the full experiment (three sessions). All had normal or corrected to normal vision and none was expert in performing arts (dance, music, mime or theatre) or in sports.

### Procedure

#### Recording sessions

In a series of three individual sessions, each one separated by at least 1 week and at most 2 months between the first and the last, participants performed a postural improvisation task in the antero-posterior direction (nine trials of 30 s per session). Inspired by the mirror game paradigm (Noy, Dekel & Alon, 2011), adapted for studying whole-body movements (Coste, Słowiński, Tsaneva-Atanasova, Bardy & Marin, 2017), participants were asked to create interesting and various postural motions by keeping their knees extended with their toes and heels in constant contact with the floor (force platform). This task enables participants to produce rhythmic postural motions at different amplitudes and frequencies. Movement kinematics was acquired using a VICON motion capture system (Nexus MX13 Vicon System) with eight infrared cameras. The 3D positions of seven reflective markers placed on the participants’ left side were recorded with a 100 Hz sampling frequency and a spatial accuracy below 1 mm. Markers were located on head (forehead), neck (C5 vertebrae) shoulder (acromion), hip (greater trochanter), knee (lateral condyle of femur), ankle (lateral malleolus) and toe (distal head of the 1st metatarsal). This set of markers served both for the generation of point-light stimuli and for computing the similarities between participants’ signatures.

#### Perceptive task

**Fig. 1:**
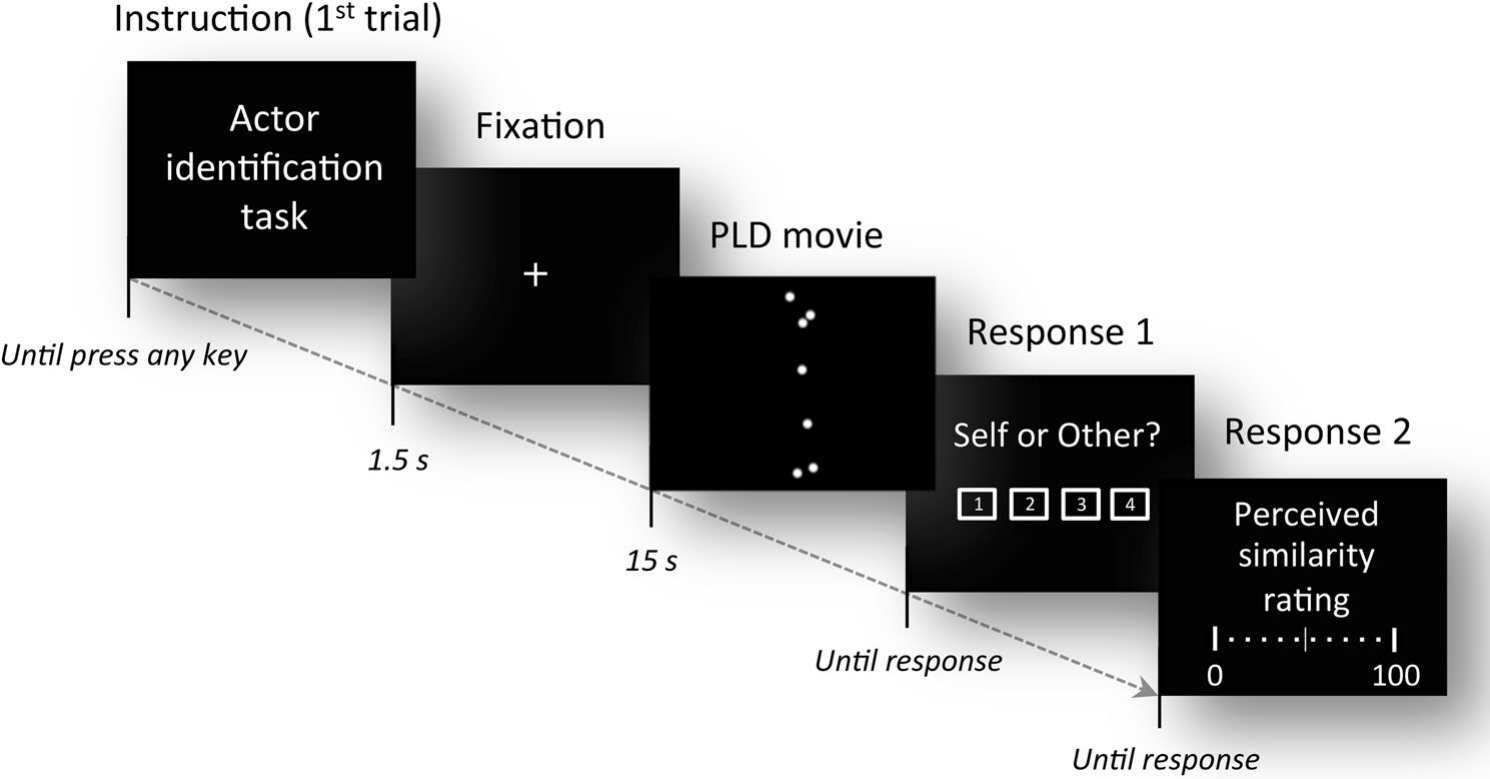
Illustration of the stimulus presentation sequence and timing

During the third and final session, immediately after the motor task, subjects performed a self-other identity discrimination task. Sitting in front of a computer screen (22-inch Dell P2213 LED monitor, resolution: 1680 × 1050 pixels, refresh rate: 60 Hz) at a distance of about 50 cm, participants viewed a total of 144 sagittal 15-s displays of point-light depictions of themselves (18 videos) and fourteen other people (14 people × 9 videos) in a random order. Trials were run in blocks of 24 videos, with 60-s break between successive blocks. Point-light displays (PLD) were produced from the recordings of the first two sessions of the same 15 subjects with Matlab R2014b (The MathWorks, Natick, MA) using the MoCap Toolbox (Burger & Toiviainen, 2013), and appeared as seven moving white dots, shape-normalized, on a black background with a 90° view angle relative to the main motion direction. The stimulus presentation sequence (Fig. 1) was also programmed in Matlab using Psychophysics Toolbox extensions (Brainard & Vision, 1997; Pelli, 1997) and consisted of a fixation period (1.5 s), a PLD movie (15 s) and two answer screens: (1) in the first one, participant was asked to judge whether the actor was himself/herself or someone else on a 4-point rating scale (1: sure other; 2: probably other; 3: probably self; 4: sure self), and (2) in the second one, participant reported a subjective measure of the perceived kinematic similarity between the actor’s movements and himself/herself, using a visual analogue scale ranging from 0 (strongly dissimilar) to 100 (strongly similar). The answer screens were presented without time limit, until the participant responded using the mouse (see SI Appendix for an illustration). No instruction was given about response time (e.g., respond as quickly as possible) to prevent any effect of time pressure on performance accuracy. Moreover, participants were not informed about the ratio between self- and other videos to prevent any response bias (i.e., the tendency to favour one response over another). Data concerning participants’ responses, response times (RTs) and mouse trajectories were all automatically stored in the computer.

### Data analysis

#### Perceptive task performance

To get a performance measure in this self/other identification task, we calculated the receiver operating characteristic (ROC) curves and their associated area under the curve (AUC) from confidence ratings (response 1). The ROC curves plot on the vertical axis the True Positive Rate (TPR) or sensitivity, i.e., the proportion of self’s trials that are correctly identified as such, and on the horizontal axis the False Positive Rate (FPR) or 1-specificity, i.e., the proportion of other’s trials that are wrongly identified as self’s trials, for all possible thresholds values. One of the main advantages of the ROC curves is its ease of interpretation. Random performance is represented by a diagonal straight line that separates the graph into two triangles. Any ROC curve in the upper left triangle indicates a good level of performance, while any ROC curve in the bottom right triangle indicates a poor level of performance. Another advantage of using the ROC curves is that the performance can be summarized with a single number between 0 and 1 by quantifying the area under the curve. An AUC score above/below 0.5 characterizes a good/bad performance, 0.5 being the AUC score of a random performance and 1 (0) the best (worst) performance.

#### Self-attribution index

A breakdown of misattributions of other productions to self-production was expressed by means of a self-attribution index. Self-attribution index ranges from 0 and 1 and represents the proportion of trials in which participant-attributed movement of another actor to him/herself (false positives). It is calculated for each of the fourteen other actors as the sum of the number of answers ‘Probably Self’ and ‘Sure Self’ for their nine videos corresponding and normalized by dividing it by nine. We also computed this index for the subject’s own trials as the sum of the number of answers ‘Probably Self’ and ‘Sure Self’ for the eighteen videos of the subject himself (true positives) and normalized by dividing it by eighteen.

#### Quantification of motor similarities

An important point of our study was to quantify the similarities between individual postural signatures using relevant metrics. One major difficulty to calculate motor similarities lied in selecting kinematic and dynamic features among a myriad of potentially relevant features (e.g., markers, joint angles, position signals and their derivatives, etc.). In addition, the selected features should, as far as possible, closely reflect those used by observers. We developed the following approach to overcome these difficulties:

1. We first calculated a perceptual distance matrix based on the similarity judgments while taking into account the reliability of judgements through the measure of performance (AUC values). Each observer (*n* = 15) gave nine perceptual judgements in the form of a similarity score *S*(*I* → *J*) ranging from 0 to 100 between his own (*I*) movements and those of the fourteen other participants (*J*). We aggregated the nine perceptual judgements for each participant to extract the median of their judgements. The perceptual similarity noted *S*_PER_(*I*, *J*) between each pair of participants was then expressed as the median similarity rating of the subject who performed best in the self-other discrimination task and collected in a matrix: If AUC (*I*) > AUC (*J*) then *S*_PER_(*I*, *J*) = *S*(*I* → *J*), otherwise *S*_PER_(*I*, *J*) = *S*(*J* → *I*). Next, we transformed the perceptual similarity matrix *S*_PER_ into a normalized perceptual distance matrix *D*_PER_. The distance matrix was obtained by first normalising *S*_PER_ into the interval [0, 1] and then *D*_PER_ = 1 − *S*_PER_. The diagonal of the distance matrix was set to zero. Note that the resulting perceptual distance matrix satisfied the axioms of a distance measure:
  Positive definiteness: *D*_PER_(*I*, *I*) = 0; *D*_PER_(*I*, *J*) ≥ 0
  Symmetry: *D*_PER_(*I*, *J*) = *D*_PER_(*J*, *I*)
  Triangle inequality: *D*_PER_(*I*, *J*) ≤ *D*_PER_(*I*, *K*) + *D*_PER_(*J*, *K*)
2. Second, we calculated a physical (trajectory) distance matrix. Specifically, we wanted to find a physical (trajectory) distance matrix that best correlated with the perceptual distance matrix. To this end, we explored combinations of several kinematic variables (position; velocity; acceleration; jerk), level of granularity (Δ*T*; pixels size) and different matrix distances measures (Frobenius distance; Manhattan L1 distance). Frobenius distance *F* is defined as: *F*_*I,J*_ = $\surd {\text{trace}}\left( {\left( {I - J} \right).\left( {I - J} \right)^{'} } \right)$, where *I* and *J* represent two matrices (images) and *J*′ is the conjugate transpose of *J*, and the Manhattan L1 distance is defined as $L_{I,J}^{1} = \mathop \sum \nolimits_{i = 1}^{n} \left| {Ii - Ji} \right|$.
  To establish optimal levels of spatial and temporal granularity, we first computed normalized spatial densities of positions’ vector for the first 15 s of the recordings as well as its various derivatives (1st: velocity, 2nd: acceleration and 3rd: jerk). To do so, the seven markers were linked to form segments. We then created normalized images (1960 × 490 mm) divided into pixels for which we counted how often segments passed into each of these pixels (number of occurrences) during the entire trial duration. Different size of pixels and time interval (Δ*T*) between two successive silhouettes were simulated. We found that a granularity of Δ*T* = 0.2 s and a pixel size of 16 mm^2^ were the best compromise between computation time and results accuracy. Next, all of the 27 images (9 trials × 3 sessions) of each participant were averaged. For each participant we computed mean images of spatial density of position/velocity/acceleration/jerk reflecting the position/velocity/acceleration/jerk explored by the participant during all of the improvisation performance. Figure 2 shows an example of the mean images of spatial density of position/velocity/acceleration/jerk, colour gradient indicates the frequency of appearance of the segments within each pixel, the red coloured pixels being the most visited places in space by the participant, while those in blue were the least visited.

**Fig. 2:**
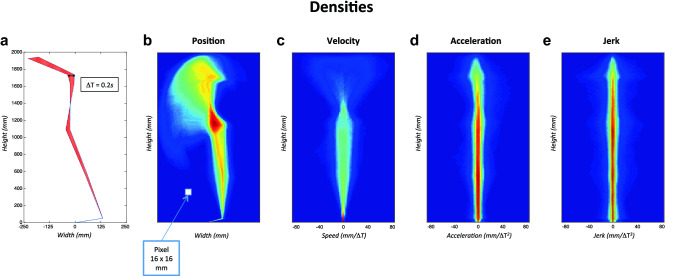
**a** Silhouette between two successive positions, with a granularity of Δ*T* = 0.2 s and a 16 mm^2^ pixel. From **b**–**e** mean densities of position, velocity, acceleration and jerk, respectively, for one participant
  To integrate information contained in the densities of position, velocity, acceleration and jerk, we compared a weighted sum of the distance matrices between the different densities. Namely, for a given set of weights *W* = [*w*1, *w*2, *w*3, *w*4], the physical (trajectory) distance matrix was expressed as follows:
  *D*_PHY_(*I*, *J*) = *w*1.*d*1(*I*, *J*) + *w*2.*d*2(*I*, *J*) + *w*3.*d*3(*I*, *J*) + *w*4.*d*4(*I*, *J*), where *d*1(*I*, *J*), *d*2(*I*, *J*), *d*3(*I*, *J*), *d*4(*I*, *J*) are the distances (calculated using the Frobenius distance or Manhattan distance) between two (*I* and *J*) densities of position, velocity, acceleration, jerk, respectively. Using a set of 1000 weights *W* combinations and the type of distance (Frobenius or Manhattan distance) as variables, we calculated 2000 correlation coefficients between the physical (trajectory) distance matrix *D*_PHY_ and the perceptual distance matrix *D*_PER_. We found that highest correlation distance (Pearson’s *r* = 0.59, *p *< 0.001; see SI Appendix for the scatterplot) was achieved using the following weight combination *w*1 = 0.5; *w*2 = 0; *w*3 = 0; *w*4 = 0.5 and the Manhattan distance. In other words, in the postural task promoting exploration and expressiveness, the perceptual similarity was best captured by combination of the position (visited places in space) and jerk (smoothness of movement trajectories).
3. Third, we used multidimensional scaling (MDS), a powerful mathematical procedure, for representing the perceptual/physical similarities of participants’ signatures visually as 2D-maps (e.g., Giese, Thornton & Edelman, 2008; Pollick, Fidopiastis, et al., 2001; Pollick, Paterson, Bruderlin & Sanford, 2001; Słowiński et al., 2016). Since MDS generates low-dimensional spaces with arbitrary scaling and axes’ orientations to compare two visual representations, it is necessary to first align them. Thus, the resulting perceptual and physical configurations were assembled in a new MDS plot by fitting the physical space to the perceptual space using a Procrustes transformation. Procrustes transformation allows to align the points of the physical space to the points of the perceptual space by applying a linear transformation (translation, reflection, rotation and scaling).
4. Finally, we performed a hierarchical clustering of both perceptual and physical distances. Hierarchical clustering analysis allowed us to objectively group participants with similar postural signatures in a data-driven manner. To interpret and assess the quality of clustering, we applied silhouette analysis (see SI Appendix for more details). These analyses were motivated by two main reasons. First, finding similar groups of participants with both distance matrices would reinforce the validity of our method for correlating perceptual and physical similarities. Second, grouping participants according to their level of similarity enable the comparison of their performance, since subjects are somewhat placed in identical conditions in terms of task difficulty. Indeed, since we have hypothesised that similarity affects performance, it is likely that performance can be influenced by the structure, location and distribution of participants’ signatures in the perceptual/physical spaces. As a result, typical postural signatures are assumed to be subject to heightened competition during recognition with the presence of many neighbouring signatures. By contrast, atypical postural signatures with fewer neighbours are likely to be exposed to less competition and are, therefore, more distinctive and more recognizable.

### Statistical analysis

To test that performance of the perceptive task differs significantly from chance level set at 0.5, we performed a one-sample *t* test taking the area under the ROC curve (AUC) as dependent variable. The effect size is reported using the Cohen’s *d* and interpreted according to Cohen’s guideline (1988), such that *d *= 0.2 corresponds to a small effect, *d *= 0.5 to a medium effect and *d *= 0.8 to a large effect. A multiple linear regression analysis was also conducted to explore the extent to which the independent variables: (1) self-perceived similarity that corresponds to the mean similarity ratings of all self-trials and (2) other-perceived similarity that corresponds to the mean similarity ratings of all other-trials; were related to the test performance (dependent variable: AUC). Finally, we performed a Pearson’s correlation between self-attribution index and similarity score ratings.

## Results

### Overall performance

**Fig. 3:**
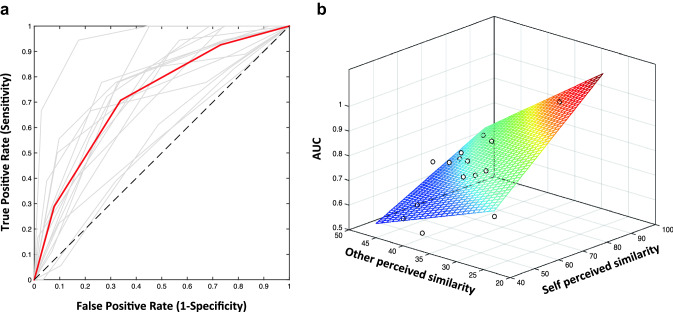
**a** Individual (in gray) and average (red) ROC curves. Dashed diagonal line corresponds to the random performance. **b** 3D plots of the perceptive task performance (AUC), the self- and other-perceived similarity score, from which a linear regression model that best fits the data points is built. Self-perceived similarity and other-perceived similarity were found to significantly predict the perceptive task performance [*R*^2^  =  0.78, *F*(2, 12)  =  21.16, *p*  < 0.001] (colour figure online)

**Fig. 4:**
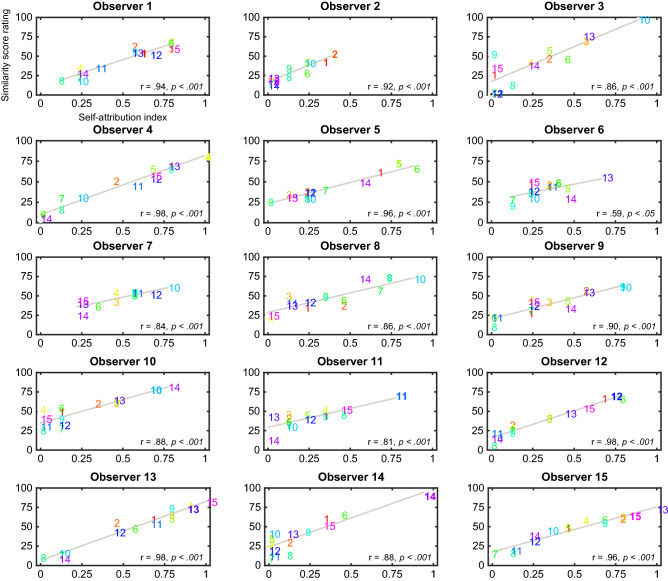
Correlations between the self-attribution index (*x*-axis) and the similarity score rating (*y*-axis) for each participant (box). Coloured numbers indicate the position of the fourteen other actors as well as the observer himself/herself (in bold) in the 2D plots. A quick reading from the right or the left of each box allows seeing the proportion of self-attributed trials according to each actor in an ascending or descending order. Pearson’s *r* coefficient and statistical significance are shown in the lower right corner of each box

The individual performances in the perceptive task (ROC curves) as well as the average performance over all participants are shown in Fig. 3a. On average, participants correctly identified their self-generated actions and those of others, since performance accuracy (mean AUC = 0.71 ± 0.11) was significantly above chance [*t*(14) = 7.87, 95% CI (0.65, 0.77), Cohen’s *d *= 2.03, *p* < 0.001]. However, one can observe substantial inter-individual variations between the best performance (AUC = 0.94) and the poorest at chance (AUC = 0.53). In particular, we noticed that some participants were unable to recognize their self-generated actions and/or attribute to themselves the other-generated actions (see Fig. 4 and SI Appendix, Fig. SI9). The Pearson’s correlation between the self-attribution index and the similarity score ratings reveals a significant positive correlation (*r *= 0.86, *p *< 0.001; Fig. SI10), suggesting that participants based their decision as to whether the observed movements were theirs or not according to the level of perceived similarity with the actor (see Fig. 4 for individual correlations and SI Appendix, Fig. SI10). Furthermore, we found that some participants (e.g., P4 and P13) adopted a very high acceptance criterion, claiming authorship for almost all videos including those of others (see response bias in SI Appendix, Fig. SI5). By contrast, some participants adopted a very low acceptance criterion (e.g., P2, P3, P6 and P10) claiming the authorship of almost none of videos, including their own productions. Such strategies can affect positively or negatively the proportion of self’s trials that are correctly identified as such, making the proportion of correct self-trials an invalid measure of sensitivity as opposed to the “bias-free” ROC analyses employed here.

To test the extent to which the independent variables: (1) self-perceived similarity; (2) other-perceived similarity; predicted the self-other discrimination performance (dependent variable: AUC), we conducted a multiple linear regression analysis. Self-perceived similarity was shown to significantly predict the test performance [*β*  =  0.84, *t*(12)  =  5.84; *p*  <  0.001], while the other-perceived similarity had similar predictive power but had opposite direction of the effect [*β*  =  − 0.67, *t*(12)  =  − 4.67; *p*  < 0.001] [Whole model: *R*^2^  =  0.78, *F*(2, 12)  =  21.16, *p*  <  0.001; Fig. 3b]. Hence, the more observers subjectively evaluated that their self-generated actions were very similar to their own usual movements and that those of others were very different, the better the self-other discrimination performance (Fig. 3b).

### Perceptual and physical spaces

**Fig. 5:**
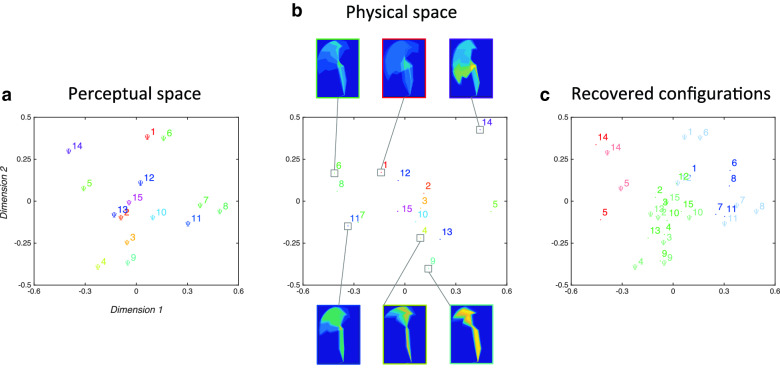
MDS plots. **a** visual perceptual space constructed by the first two dimensions of MDS and based on judgments of kinematic similarity. Phi symbols (Ψ) indicate the position of the participants’ postural signatures into the psychological space. **b** Physical space constructed by the first two dimensions of MDS and based on the physical trajectory distance. Dots indicate the position of the participants’ postural signatures into the physical space. Densities of position for representative trials are also shown. **c** Resulting MDS map for perceptual and physical distances after Procrustes alignment. The three distinct groups of participants obtained by hierarchical clustering are represented in three different colours (opaque colours for the perceptual space and default colours for the physical space)

It is possible that some movement styles, very distinctive, have been more favourable than others for recognition. To test this hypothesis and assess the similarities between participant’s signatures, we constructed the perceptual (subjective) and physical (objective) spaces by applying multidimensional scaling to perceptual judgements and physical trajectories. Figure 5 shows the resulting MDS plots. In these abstract spaces, the similarity/dissimilarity between participant’s motor signatures is represented by the distance between geometric point representations. Strikingly, we show that the physical space—constructed from distances between the weighted combinations of position and jerk densities—resembles the configuration of the perceptual space after a Procrustes transformation step (Fig. 5c). This result is consistent with that of Giese et al. (2008), and supports the idea that the perceptual representations of biological motion is veridical and closely reflects physical movement trajectories.

### Hierarchical clustering

Hierarchical clustering analysis provided further insights into the similarities between the postural signatures. In particular, we found that at a level of precision of three clusters, the two (perceptual and physical) distances’ matrices gave the same clusters of participants except for one element (participant 12). The silhouette analysis reveals a good quality of the clustering (see SI Appendix for a detailed analysis).

Finally, comparing visually the performance across groups of participants with similar signatures (clusters), it can be observed that the variation in performance within groups is smaller than the variation between groups (SI Appendix, Fig. SI8), suggesting that individual differences in identity perception ability relies more on individual variations in kinematic patterns rather than on differences in visual processing.

## Discussion

In the present study, we investigated whether and to what extent motor similarities between people have an impact on the ability to discriminate self/other-generated actions. We first recorded the idiosyncratic motion variability (i.e., individual postural signatures) of fifteen individuals in a postural improvisation task and created from this a wide range of diverse set of point-light depictions. We then examined the self/other discrimination performance of the same fifteen individuals and sought to relate this performance to the metric properties of perceptual/physical representations of postural signatures. Our results show that identity perception ability varies substantially across individuals and is partly related to the perceptual/physical motor similarities between self and other PLD stimuli. In particular, we found that the movement patterns generated by people have a kind of “kinematic fingerprints”, and that some of these motor traces are easier to decode than others, since they are more distinctive.

As a main finding of this study, we found that participants improvising postural movements can well recognize, on average, their own productions even after several weeks. This suggests that individual postural signatures are sufficiently distinctive and persistent over time to be recognizable. We should note, however, that large differences in performance levels were also found between those who excelled in recognizing their own past actions and who had random performance. More specifically, the similarities with other participants’ actions were notoriously error prone and led to many misidentifications. It seems that participants based their decision as to whether the observed movements were theirs or not according to the level of perceived similarity with the actor, as evidenced by the strong correlation between perceptual judgments and the self-attribution index. Accordingly, participants guessed themselves when the perceived similarity between produced and observed actions was high. When the perceived similarity between produced and observed actions was very low, participants guessed themselves less often. In contrast, when PLD movies were neither strongly similar nor very dissimilar, the self/other discrimination was not as successful. Participants were generally less confident in their responses what was reflected in much longer response times and larger number of abrupt shifts between responses categories along the horizontal axis (see individual response times and *x*-flips shown in Figs. SI2 and SI3, SI Appendix). Our results are also consistent with the similarity principle inherent in the common coding theory (Prinz, 1997; Hommel et al., 2001): the more similar an observed action is to the way the observer would perform the very same action, the higher the activation of common codes; and provide further evidence in favour of the contribution of the motor system to identity perception (e.g., Casile & Giese, 2006; Knoblich & Sebanz, 2006; Loula et al., 2005; Su & Keller, 2018). For instance, Calvo-Merino et al. (2005) found a greater activation of the mirror system when ballet dancers and capoeira dancers watched the type of dancing, they were experts in (i.e., that with which they had a great motor experience). Importantly, a greater activation was observed even though it was not their own dance movements. In this respect, our results highlight the problem of misattribution (e.g., Jeannerod, 2003; Schütz-Bosbach & Prinz, 2007) when motion similarities (and the activation of common codes) between two individuals are sufficiently high. Surprisingly, some participants failed to identify their own past actions, but rather attributed to themselves the other-generated actions with signatures that were sometimes very different from their own. One reason why they failed in self-recognition may be the discrepancies between the motor representations acquired through action execution and the feature representations obtained during action observation. This potential explanation is supported by studies showing that we use mappings developed from previous experiences, such that visual perception sensitivity increases with the visual and/or motor experience (e.g., Bläsing & Sauzet, 2018; Casile & Giese, 2006; Loula et al., 2005). In line with this perspective, it is likely that these observers need more experience to appreciate subtle differences between their own actions and those of others. Indeed, we noticed that all the participants concerned were assigned in two of the three clusters, those whose movement patterns were the most similar (i.e., with less distinctive signatures) and, therefore, harder to recognize. Another possibility of self-identification failure, which is also related to the previous one, may be due to motion variability. Whether intra-individual (i.e., carrying out different actions; inter-trials variations) or inter-individual (dissimilarity from person to person), the variability can affect categorization and similarity judgments (Rips, 1989). Once again, self-recognition performance may benefit from perceptual experience to cope with the real-world variability. Future researches should thus explore more broadly how the perceptual experience of self-generated actions, other-generated actions and performance evolves over time.

As a second point of interest, we investigated how perceptual and physical representations of postural signatures were related. To date, the relation between perceptual and physical representations has not been extensively explored in the biological motion perception literature (see Giese et al., 2008; Pollick, Fidopiastis, et al., 2001; Pollick, Paterson, et al., 2001). However, relevant insights can be drawn from the literature on the perception of the faces and voices, given the several compelling similarities proposed between processing of body motion, faces and voices (for reviews see Yovel & Belin, 2013; Yovel & O’Toole, 2016). In particular, it has been argued that faces and voices identities are encoded in multidimensional perceptual spaces (“face-space” and “voice-space”, respectively). The dimensions of these spaces are assumed to correspond to information that people use to discriminate faces/voices and the distance between identities representations to reflect the degree of similarity between faces/voices. The origin of these spaces is supposed to represent the average of all faces/voices experienced by a person. Thus, the more typical faces/voices are, the closer they are to the origin of these spaces and the more distinctive the faces/voices are, the further away they are. Remarkably, this similarity-based framework accounts for a wide range of face and voice recognition phenomena, such as the face inversion effect, caricature effects and distinctiveness effects (e.g., Valentine, 1991; Latinus et al., 2013; Lee et al., 2000). In biological motion, several studies have uncovered very similar phenomena at a behavioural level (e.g., Hill & Pollick, 2000; Giese et al., 2008; Pollick, Fidopiastis, et al., 2001; Sumi, 1984). For example, it has been evidenced that exaggerate physical properties of biological motion such as temporal (Hill & Pollick, 2000) or spatial (Pollick, Fidopiastis, et al., 2001) information made stimuli more discriminable, suggesting that physical properties are psychologically important for recognition. Subsequently, Giese et al. (2008) demonstrated that visual perceptual representations of complex spatiotemporal patterns of motion (actions) conveyed by the point-light displays faithfully reflect motion trajectories of the physical world. Our current results complement and expand these previous findings by showing that recognition is essentially based on salient motion properties that are both stable over time and distinctive between individuals. Moreover, we show for the first time that observers are sensitive to the individual-specific patterns of movement (i.e., individual motor signatures) and that the visual perception of these signatures is veridical in the sense that closely reflects the physical trajectories of postural movements. The strong correlation between perceptual judgments and the weighted combination of physical trajectories cues as well as the output of hierarchical clustering lead us to conclude that both position and jerk kinematic cues were the most salient information contained in biological movement patterns used by at least the most decisive participants in the definition of the perceptual matrix and the resulting clusters. This does not mean, however, that observers did not use the velocity and acceleration cues for recognition, but probably that these cues provide redundant information with the position and jerk cues given that kinematic properties are highly correlated with each other (Pollick et al., 2002). In addition, it has been shown that human observers are generally less effective than computer algorithms in performing such a task, indicating that people exploit only a portion of available information in point-light biological motion (Pollick et al., 2002).

Finally, we believe that the study of the issue of similarity on recognition is a good starting point for fostering the dialogue between the two current parallel lines of research in psychology (visual perception) and biometrics (computer vision and pattern recognition), which would mutually benefit from each other for a deeper understanding under what conditions and tasks are required for an effective recognition. Moreover, this study constitutes a first step in addressing the issue of similarity and the inter-individual differences in a point-light identity recognition accuracy task. Further work should explore the similarity effect in biological motion of other socially driven cues such as gender, actions, intentions or emotions. For instance, in the case of emotional signatures, motion patterns associated with surprise and happiness, compared to those associated with sadness, are less distinctive and might be more likely to be confused (Coulson, 2004).

## Electronic supplementary material

Below is the link to the electronic supplementary material.

Supplementary material 1 (PDF 5187 kb)

Supplementary material 2 (MP4 2521 kb)

## Data Availability

Data are not publicly available due to their containing information that could compromise the privacy of research participants. The datasets and code generated during the current study are, however, available from the corresponding author on request.
