# Supplementary material for: Decoding identity from motion: how motor similarities colour our perception of self and others
Source: Psychol Res. 2020 Feb 6;85(2):509–19. doi: 10.1007/s00426-020-01290-8 (PMC7900038; doi:10.1007/s00426-020-01290-8)
Supplement: Supplementary file 1 — Supplementary material 1 (PDF 5187 kb) [file 426_2020_1290_MOESM1_ESM.pdf]

Supplementary Information Appendix for « Decoding identity from motion: How motor similarities colour our perception of self and others»

Authors: Alexandre Coste*, Benoît G. Bardy, Stefan Janaqi, Piotr Słowiński, Krasimira Tsaneva-Atanasova, Juliette Lozano Goupil & Ludovic Marin

Table of Contents

- *Figure SI1: Response screens of the Psychtoolbox interface*
- *Figure SI2: Individual response times (RTs) in seconds according to the level of perceived similarity between the participant and the actor*
- *Figure SI3: Number of x-flips according to the level of perceived similarity between the participant and the actor*
- *Figure SI4: Individual ROC curves in color and their associated area under the curve (AUC)*
- *Figure SI5: Response bias (c) for each participant*
- *Figure SI6: Correlation between the perceptual distance matrix and the physical (trajectory) distance matrix*
- *Figure SI7: Hierarchical clustering dendrograms and silhouettes analysis*
- *Figure SI8: Boxplots of the resulting three clusters*
- *Figure SI9: Self-attribution index and perceived similarity ratings according to the different actors/participants*
- *Figure SI10: Correlation between the self-attribution index and the similarity score ratings*
- *Figure SI11: Position density images of one participant across the 3 sessions and the corresponding average density position*

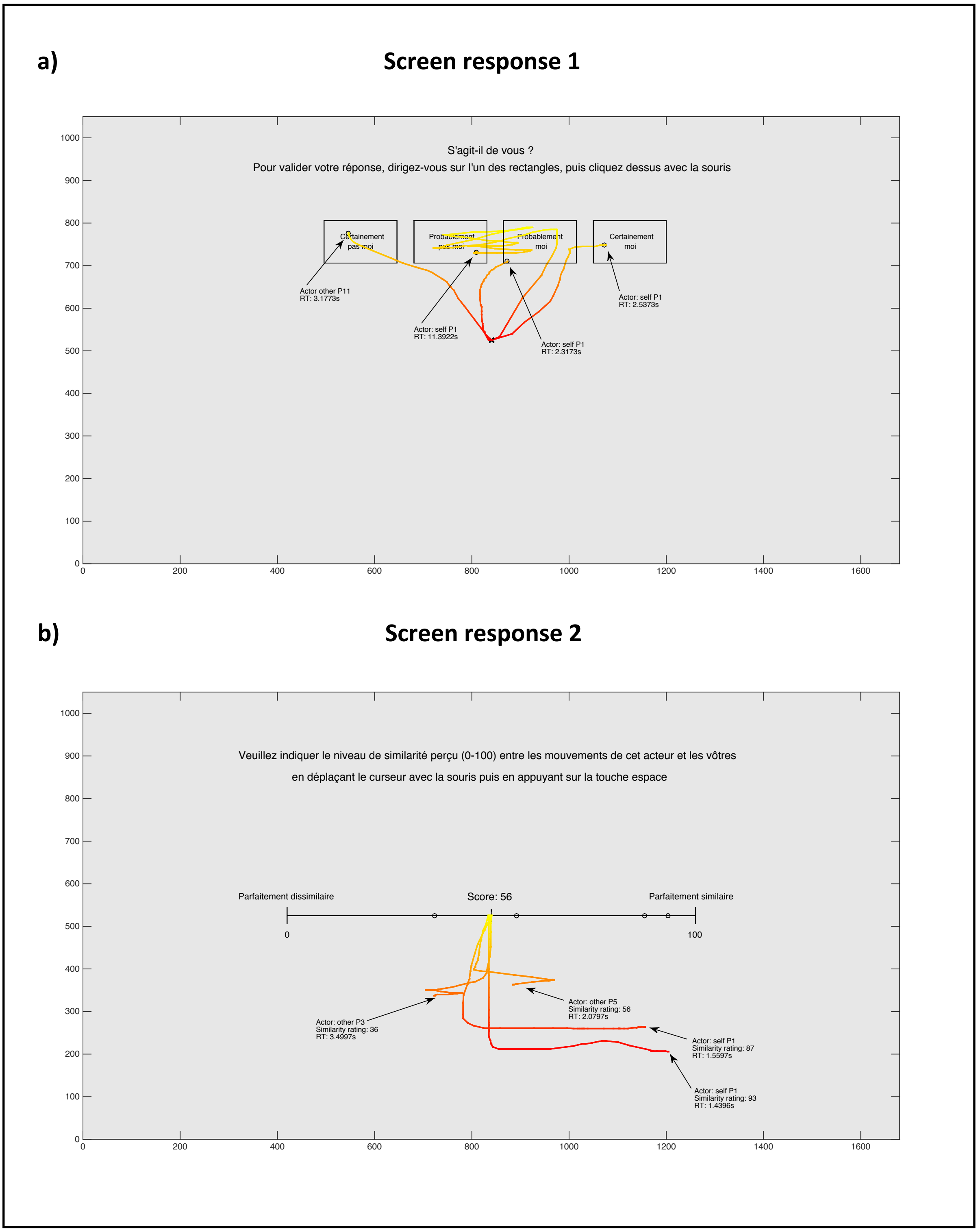

Figure SI1: Top: 1st response screen of the Psychtoolbox interface. Participants were instructed to answer the question: “Is it you?” according to a 4-point scale using the mouse. The mouse trajectory, the chosen response, the response time (RT) and the actor’s identity of the PLD movie are shown on the figure a). Bottom: 2nd response screen of the Psychtoolbox interface. Participants were asked to indicate the degree of perceived similarity between the actor's movements and their own. The mouse trajectory, the similarity score, the response time (RT) and the actor’s identity of the PLD movie are shown in the figure b).

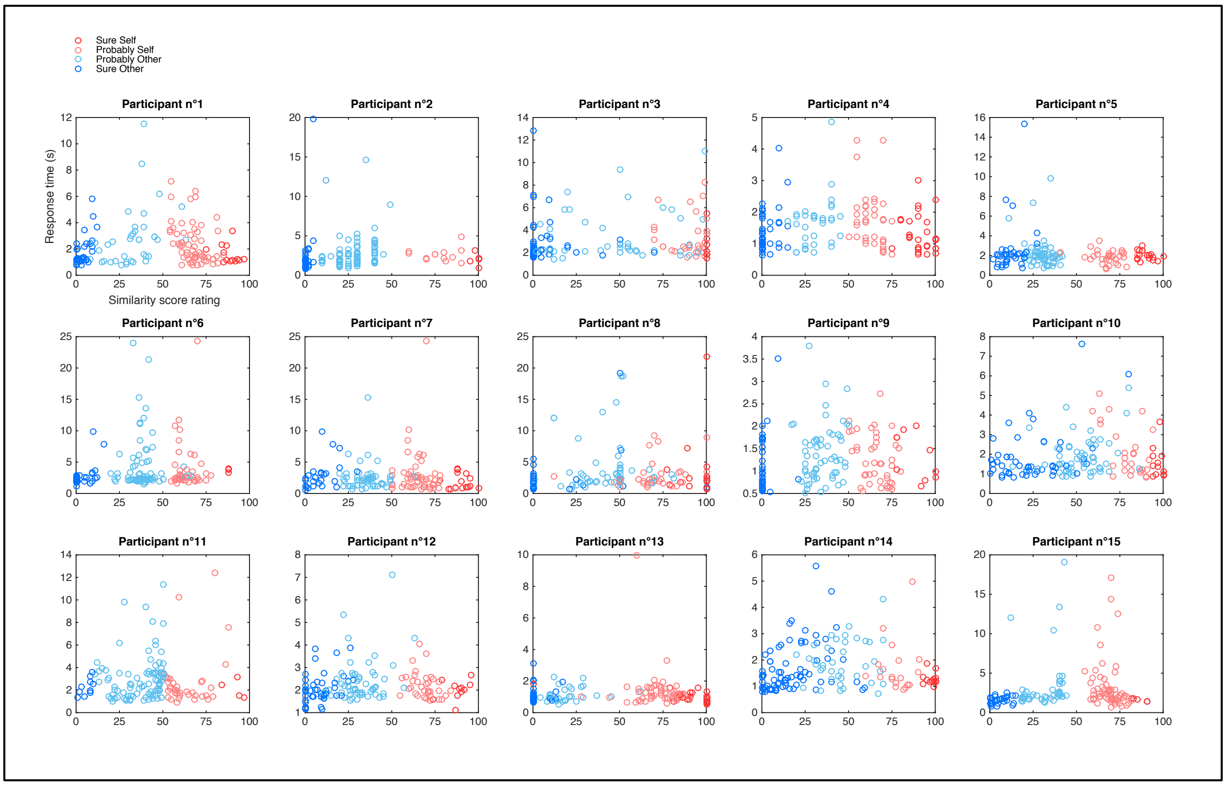

Figure SI2: Individual response times (RTs) in seconds according to the level of perceived similarity between the participant and the actor. Response time (RT) was calculated as the time interval between the onset of the display of 1st response screen and the time when the participant clicked with the mouse to answer it. Coloured circles indicate the participant' answer on the 4-pt rating scale (1/blue: sure other; 2/light blue: probably other; 3/light red: probably self; 4/red: sure self). For the sake of visibility, 2D plots have different y-axes ranges.

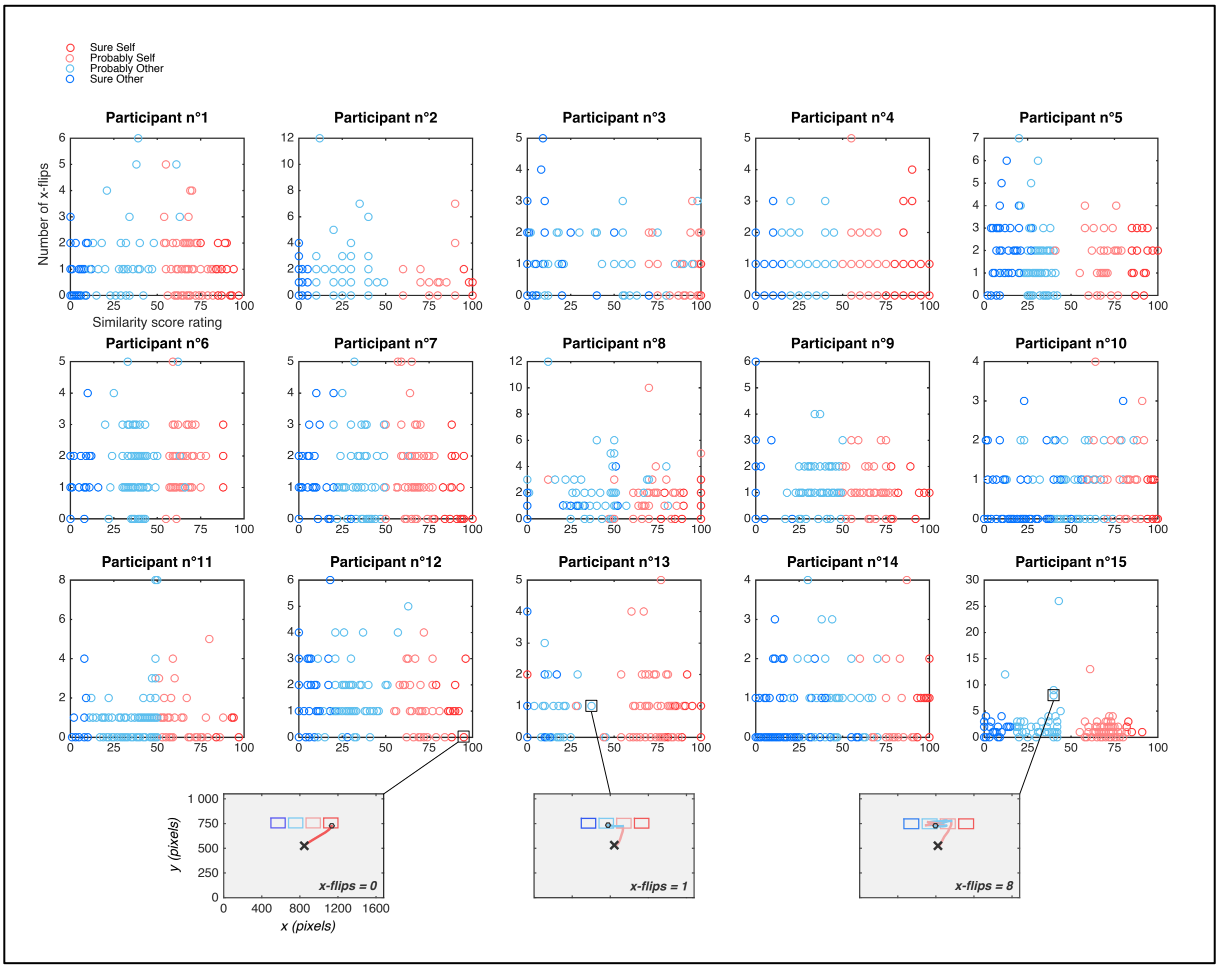

Figure SI3: Number of times the mouse cursor changes direction along the *x*-axis (i.e., *x*-flips) according to the level of perceived similarity between the participant and the actor. Three representative mouse trajectories and their corresponding number of *x*-flips (zero times, one times and eight times in the given example trajectories) are shown at the bottom of the figure. Coloured circles indicate the participant' answer on the 4-pt rating scale (1/blue: sure other; 2/light blue: probably other; 3/light red: probably self; 4/red: sure self). For the sake of visibility, 2D plots have different y-axes ranges.

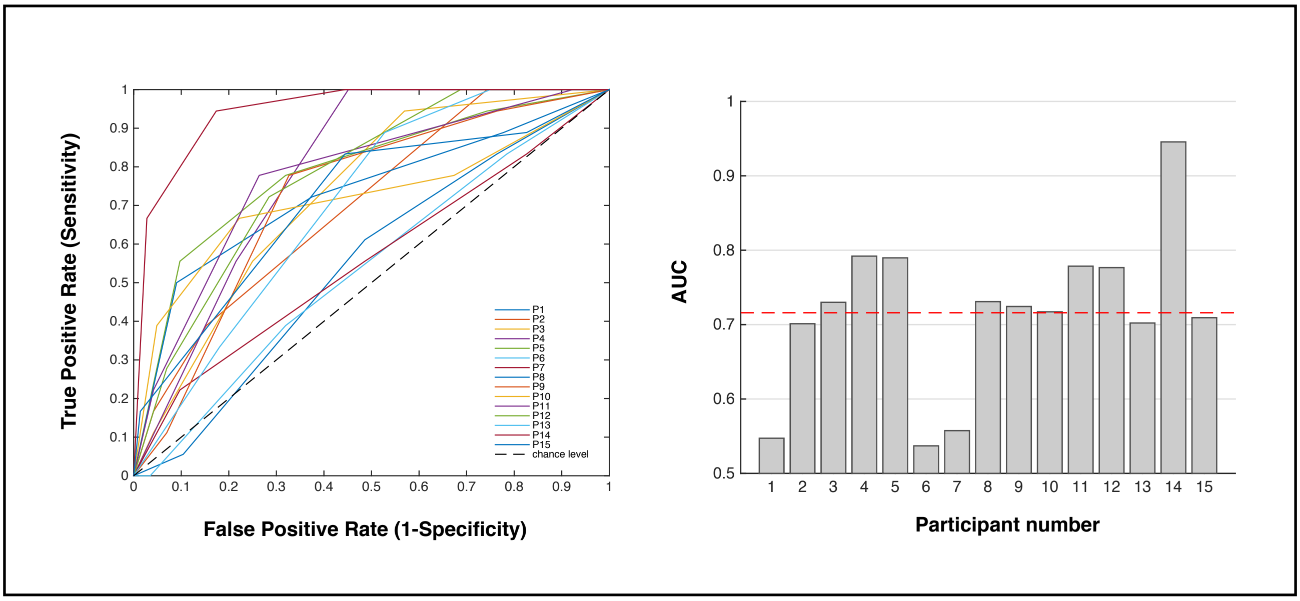

Figure SI4: Individual ROC curves and their associated area under the curve (AUC). Dashed lines correspond respectively to the random performance (left) and the mean performance across participants (right).

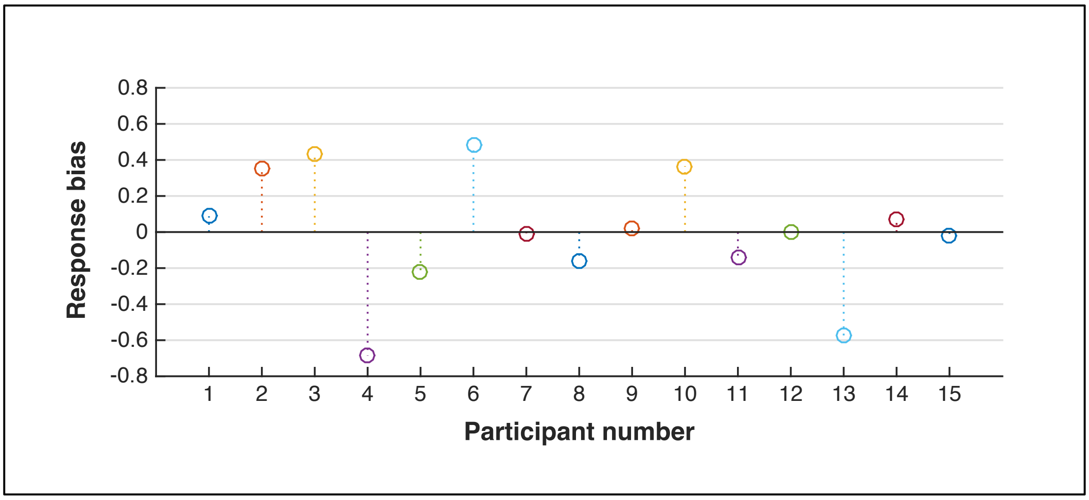

Figure SI5: Response bias (*c*) for each participant (according to Macmillan & Creelman, 2005). Negative values of *c* indicate a bias toward responding "yes" (Self), rather than "no" (Other) to the question: Is it you? and conversely for positive values. Unbiased criterion corresponds to a value of *c* equal to 0.

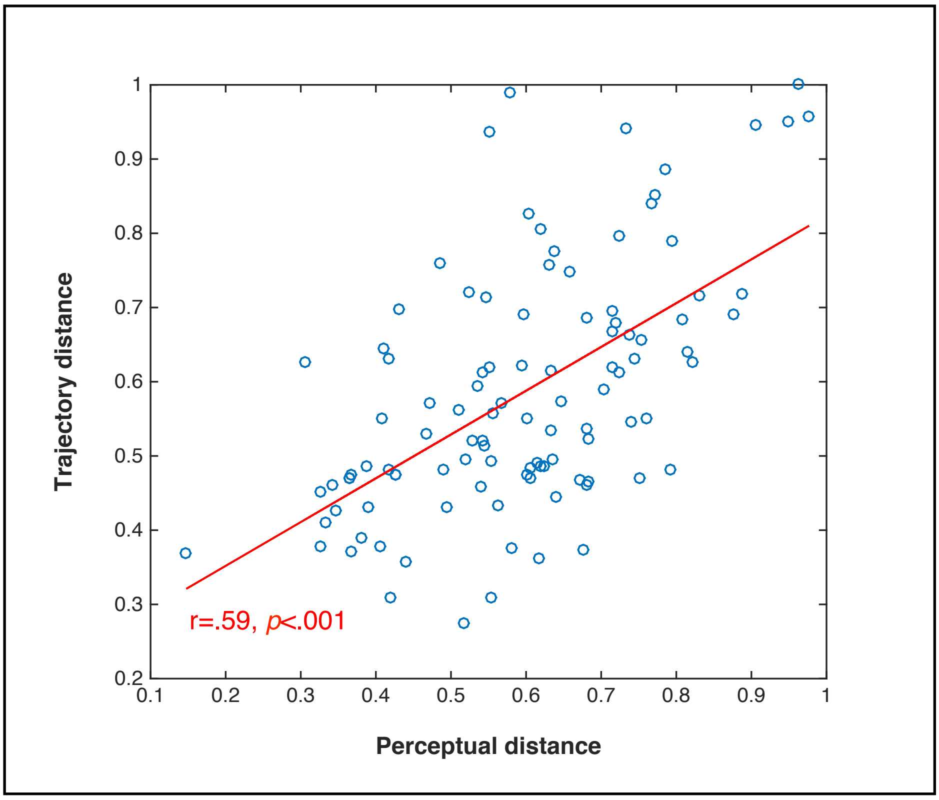

Figure SI6: Correlation between the perceptual distance matrix and the physical (trajectory) distance matrix calculated for a set of weights $w1 = 0.5$; $w2 = 0$; $w3 = 0$; $w4 = 0.5$ and using the Manhattan distance. Weighting coefficients *w1*; *w2*; *w3*; *w4* correspond respectively to densities of position, velocity, acceleration and jerk. Since the diagonals of the two distance matrices are equal to zero, we have excluded them for the calculation of the correlation and show on the figure only the couples whose values are different from zero (blue circles). Pearson's $r_{per,traj}$ coefficient is equal to 0.59 ($p<0.001$).

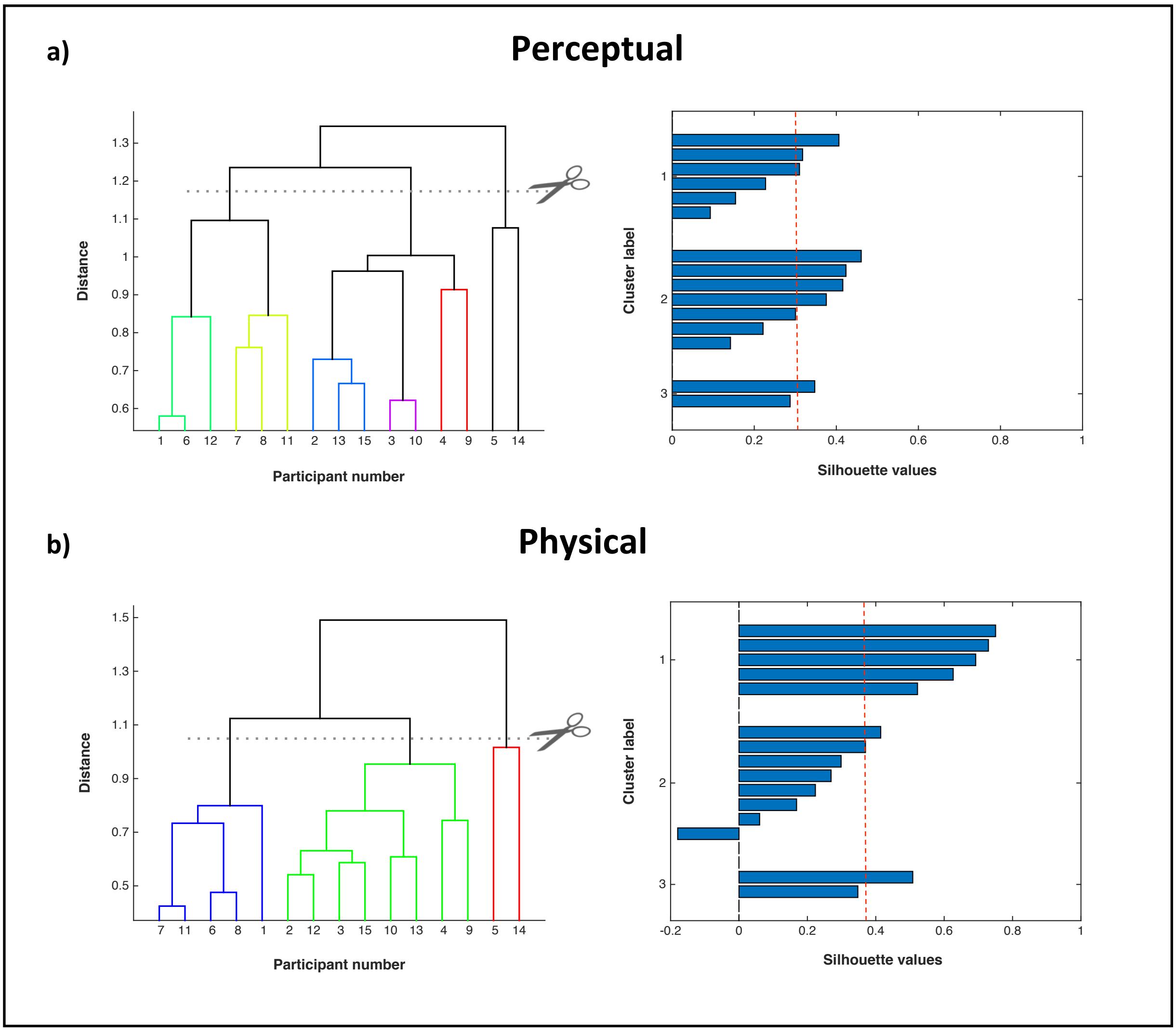

Figure SI7: Left: Hierarchical clustering dendrograms of the perceptual distance matrix (a) and the physical distance matrix (b). The horizontal axis represents participants and clusters. The vertical axis represents the distance between the clusters. The black dashed line indicates the optimal number of clusters found using a clustering quality measure (silhouette values). The number of clusters that maximizes the number of well-clustered individuals for both clustering (perceptual and trajectory) was three. Right a) and b): Silhouette plots. Silhouette value for each participant varies from -1 to +1 and is a measure of how similar that participant is to participants in its own cluster versus participants in other clusters. The red dashed line indicates the average silhouette values. The silhouette plots show a good clustering, except for one element (participant 12).

Clusters of participants obtained from the perceptual distance matrix D_{PER}:

C1 = [1 6 7 8 11 12];

C2 = [2 3 4 9 10 13 15];

C3 = [5 14];

Clusters of participants obtained from the physical distance matrix D_{PHY}:

C1 = [1 6 7 8 11];

C2 = [2 3 4 9 10 12 13 15];

C3 = [5 14];

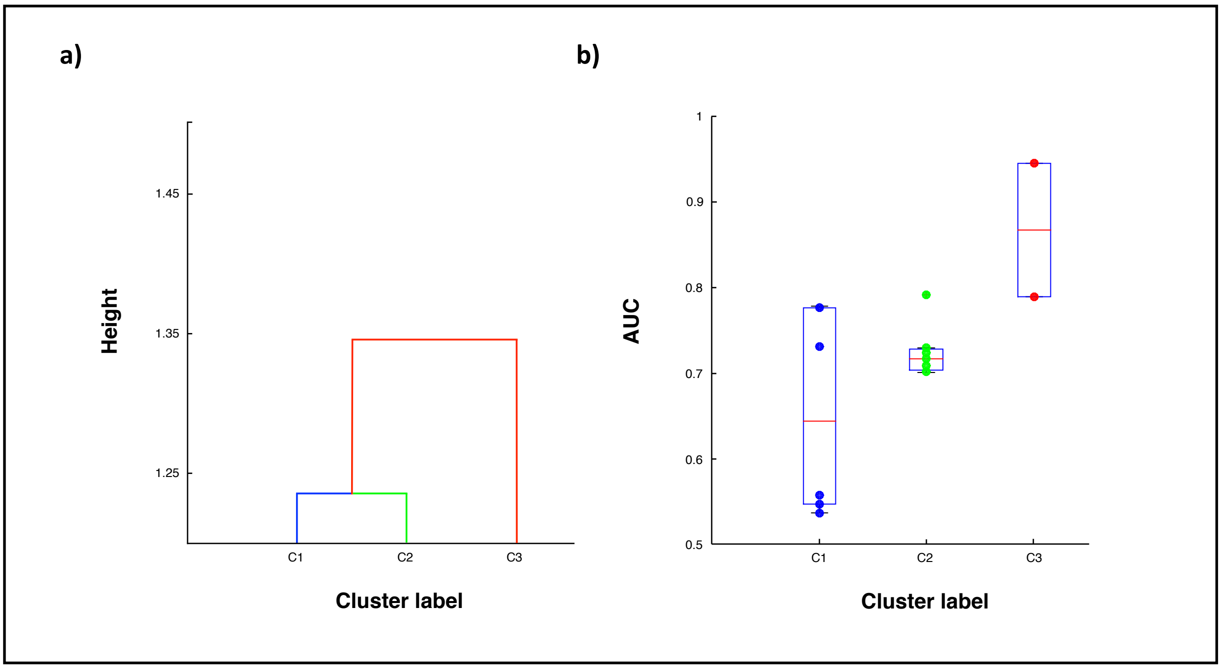

Figure SI8: a) Dendogram of perceptual distance matrix according to the three distinct clusters. b) Boxplots with individual data points of the three clusters computed by the perceptive task performance (AUC). Boxplots show median values (horizontal red line), interquartile range (box outline), minimum and maximum values of the upper and lower quartiles (whiskers) and outlier (data point outside the box).

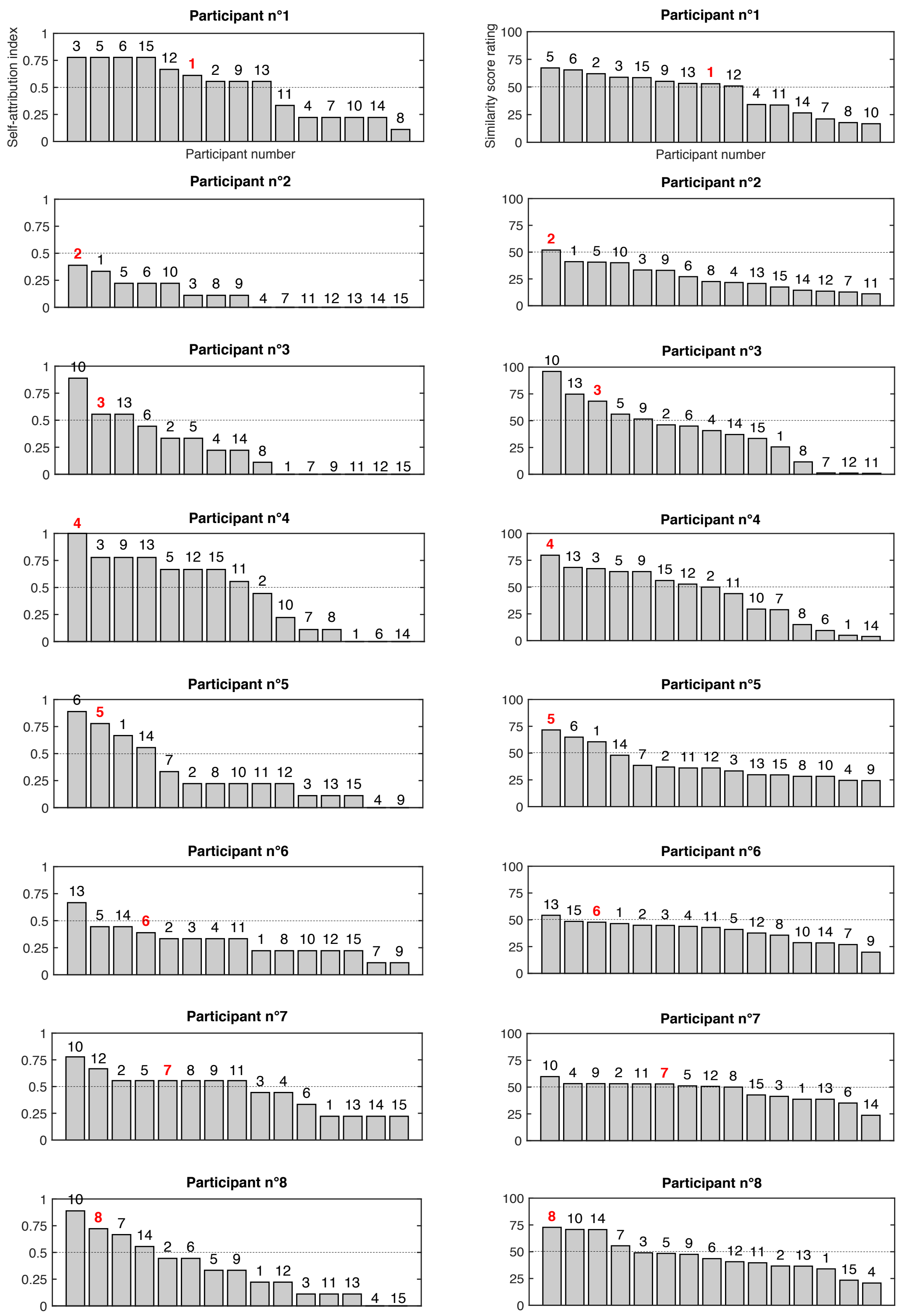
Participant n°1
Self-attribution index
Participant number
3 5 6 15 12 1 2 9 13 11 4 7 10 14 8
Participant n°1
Similarity score rating
Participant number
5 6 2 3 15 9 13 1 12 4 11 14 7 8 10
Participant n°2
2 1 5 6 10 3 8 9 4 7 11 12 13 14 15
Participant n°2
2 1 5 10 3 9 6 8 4 13 15 14 12 7 11
Participant n°3
10 3 13 6 2 5 4 14 8 1 7 9 11 12 15
Participant n°3
10 13 3 5 9 2 6 4 14 15 1 8 7 12 11
Participant n°4
4 3 9 13 5 12 15 11 2 10 7 8 1 6 14
Participant n°4
4 13 3 5 9 15 12 2 11 10 7 8 6 1 14
Participant n°5
6 5 1 14 7 2 8 10 11 12 3 13 15 4 9
Participant n°5
5 6 1 14 7 2 11 12 3 13 15 8 10 4 9
Participant n°6
13 5 14 6 2 3 4 11 1 8 10 12 15 7 9
Participant n°6
13 15 6 1 2 3 4 11 5 12 8 10 14 7 9
Participant n°7
10 12 2 5 7 8 9 11 3 4 6 1 13 14 15
Participant n°7
10 4 9 2 11 7 5 12 8 15 3 1 13 6 14
Participant n°8
10 8 7 14 2 6 5 9 1 12 3 11 13 4 15
Participant n°8
8 10 14 7 3 5 9 6 12 11 2 13 1 15 4

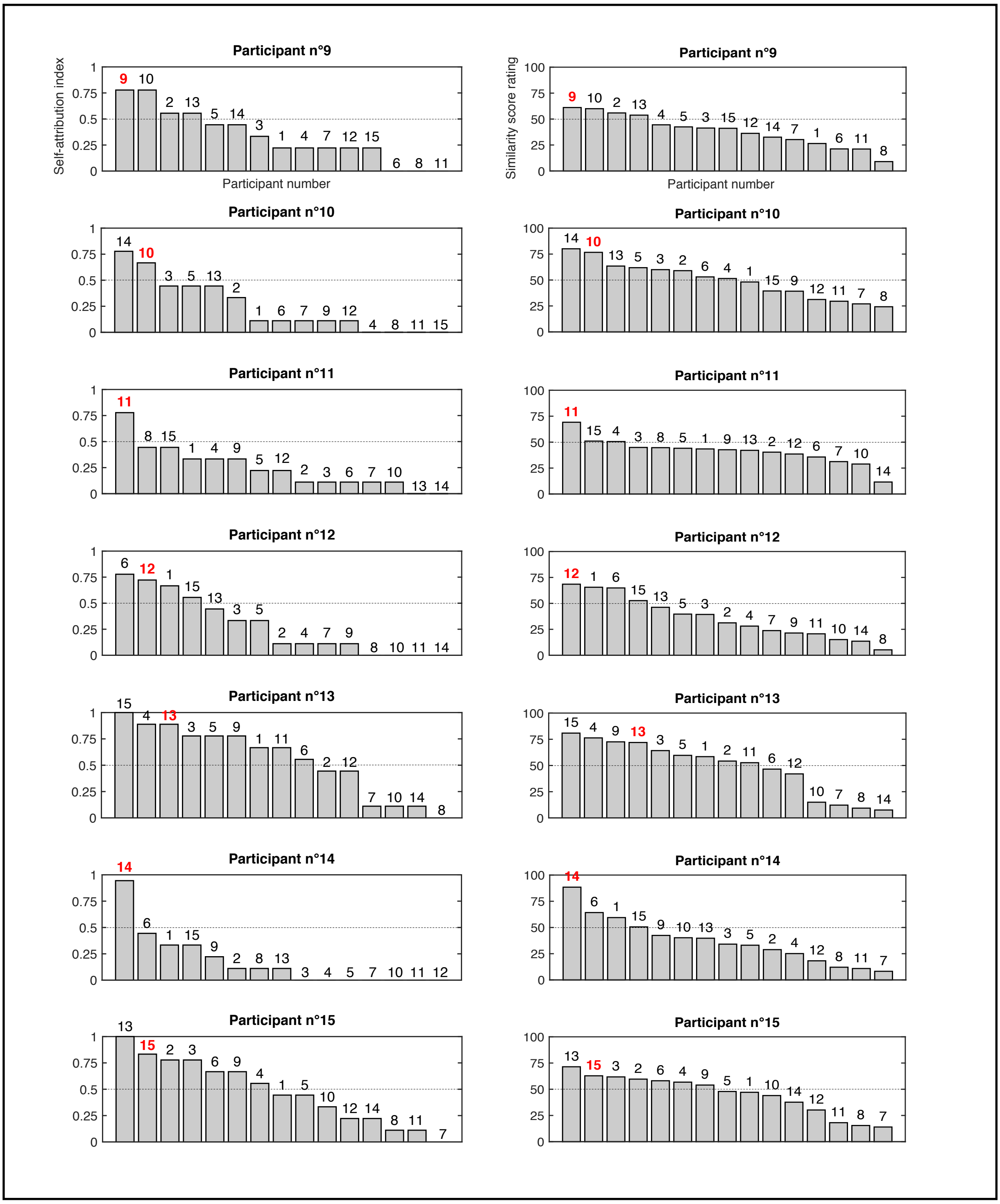

Figure SI9: a) Left: Self-attribution index according to the different actors/participants ranked in a descending order. Dashed horizontal lines indicate chance performance levels. b) Right: Mean perceived similarity ratings (resemblance) with the different actors/participants ranked in a descending order. Dashed horizontal lines indicate a similarity score equal to 50.

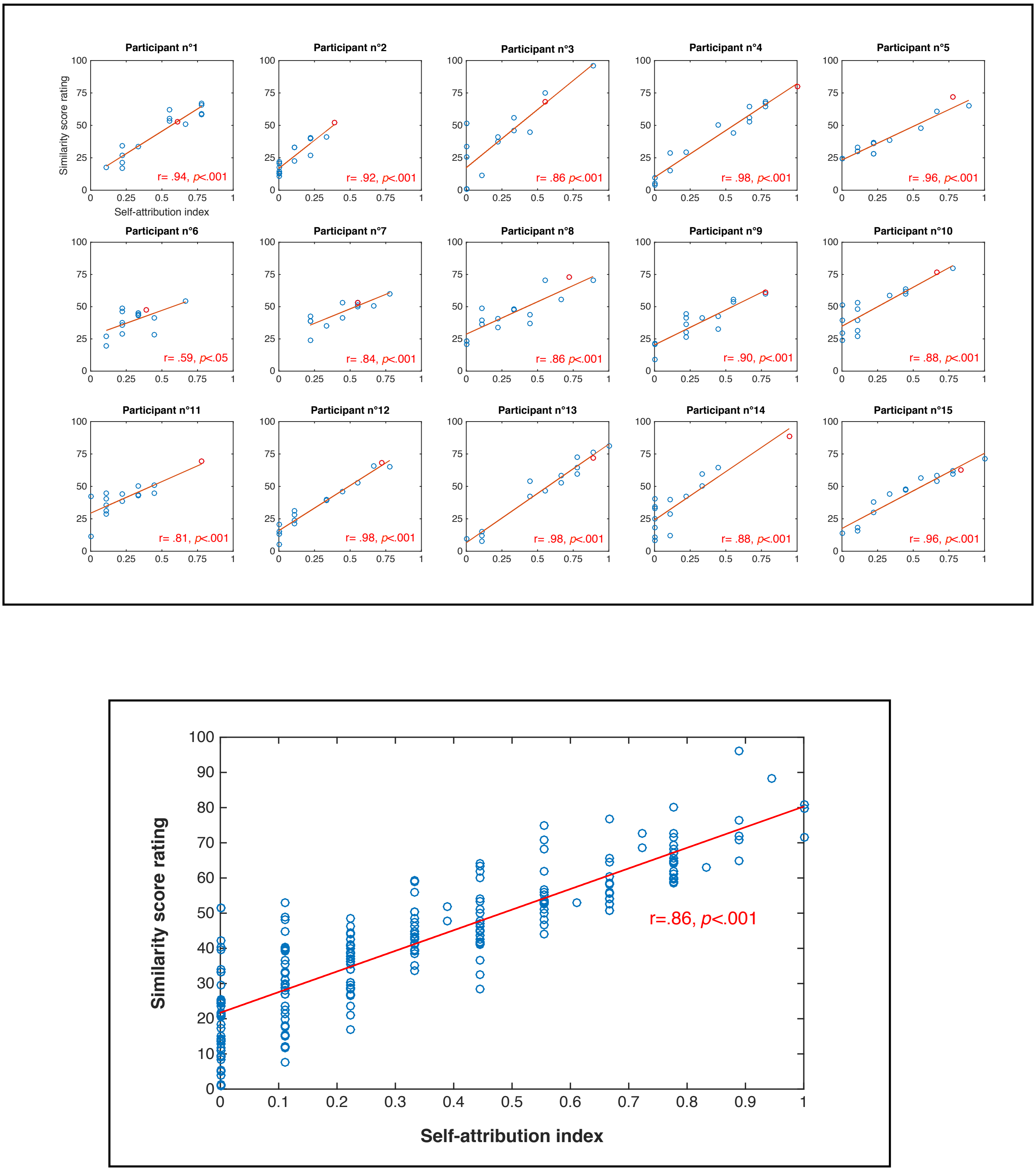

Figure SI10: Top: Correlation between the self-attribution index (*x*-axis) and the similarity score rating (*y*-axis) for each participant. Blue circles correspond to the 14 others actors. Red circle corresponds to self-trials. Bottom: Correlation between the self-attribution index (*x*-axis) and the similarity score rating (*y*-axis) for all participants. Pearson's r coefficient is equal to 0.86 ($p<0.001$).

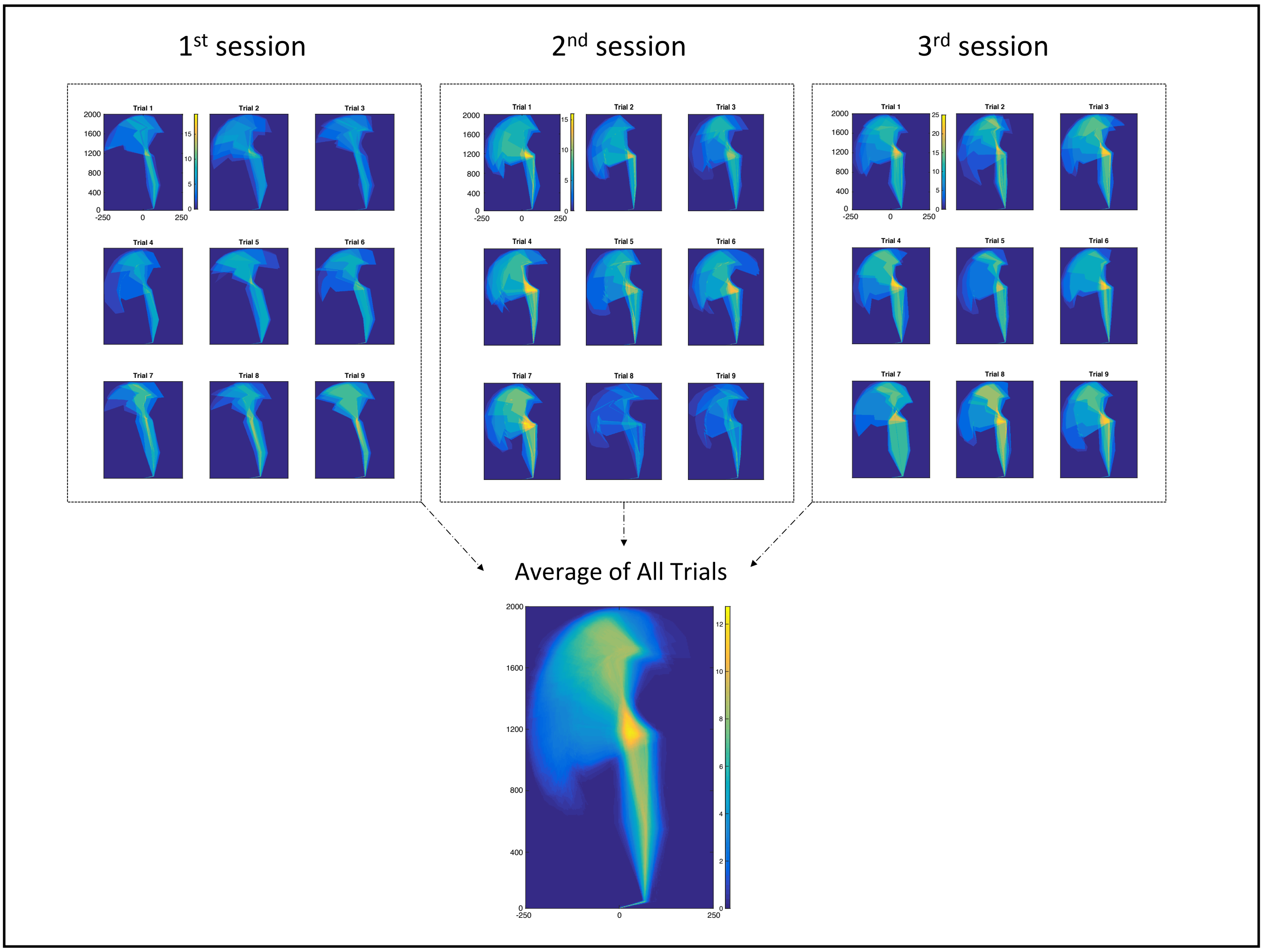

Figure SI11: Position density images of all trials of participant 12 across the 3 sessions and the corresponding average position density, somewhat illustrating his "kinematic fingerprint".
